# 超声辅助萃取-凝胶渗透色谱净化结合超高效液相色谱-高分辨质谱检测食用鱼肉中12种卤代有机污染物

**DOI:** 10.3724/SP.J.1123.2023.12028

**Published:** 2025-01-08

**Authors:** Yizhe ZHU, Ruifen ZHENG, Zihao FAN, Ling LIU, Jingyao YE, Kai WANG, Caiming TANG

**Affiliations:** 1.东莞理工学院生态环境工程技术研发中心, 广东 东莞 523808; 1. Research Center for Eco-Environmental Engineering, Dongguan University of Technology, Dongguan 523808, China; 2.中国科学院广州地球化学研究所,广东省环境资源利用与保护重点实验室, 广东 广州 510640; 2. Guangdong Key Laboratory of Environmental Resources Utilization and Protection, Guangzhou Institute of Geochemistry, Chinese Academy of Sciences, Guangzhou 510640, China

**Keywords:** 凝胶渗透色谱, 超高效液相色谱-高分辨质谱, 卤代有机污染物, 食用鱼肉, gel permeation chromatography (GPC), ultra performance liquid chromatography-high resolution mass spectrometry (UPLC-HRMS), halogenated organic pollutants (HOPs), edible fish

## Abstract

卤代有机污染物(HOPs)因具有持久性、生物积累性、毒性等而广受关注。本研究应用超声辅助萃取和凝胶渗透色谱净化结合超高效液相色谱-高分辨质谱,建立了同时检测常见食用鱼肉中12种HOPs的方法。根据HOPs特性和鱼肉样品特点选择高效、可靠的前处理方法,优化了萃取净化方法和色谱-质谱参数。鱼肉通过甲醇-乙腈(1∶1, v/v)混合溶剂提取,低温高速离心,上清液经氮吹浓缩后转溶于乙酸乙酯-环己烷(1∶1, v/v)混合溶剂,过滤后由凝胶渗透色谱净化,洗脱液经氮吹浓缩后复溶,进样分析。液相色谱流动相为含2 mmol/L乙酸铵的水溶液和乙腈,通过梯度洗脱,离子源为电喷雾电离源,负离子模式,质谱数据采集方式为全扫描和数据依赖采集,同位素内标法定量。 结果显示,12种HOPs均具有良好的线性关系,定量限为0.5 ng/g。以混合鱼肉为基质,在4、40和400 ng/g加标水平下,12种HOPs的加标回收率为67.6%~133.8%, RSD为0.5%~15.6%。应用本方法对27份实际鱼肉样品进行检测,发现鱼肉中均含有HOPs,含量为未检出~4.52 ng/g。该方法灵敏度好,准确度高,可作为鱼肉中HOPs的高通量筛查和定量检测方法。

## Abstract (en)

Halogenated organic pollutants (HOPs) have attracted considerable attention owing to their persistence, bioaccumulation, and toxicity. The development of methods to detect HOPs in fish is challenging owing to the compositional complexity of fish matrices, which contain high levels of lipids and relatively low concentrations of HOPs. In addition, the lipophilicity of most HOPs renders their extraction difficult. Moreover, the simultaneous determination of multiple HOPs to achieve the high-throughput screening of these analytes is complex.

In this study, a reliable and efficient pretreatment method based on ultrasound-assisted extraction, gel permeation chromatography purification, and ultra performance liquid chromatography-high resolution mass spectrometry (UPLC-HRMS) was developed for the determination of 12 HOPs in edible fish. The procedures of sample extraction and purification and LC-HRMS detection parameters were optimized to improve the performance of the method. Fresh fish samples were thoroughly rinsed with water, and non-edible parts, including the skin, bones, and phosphorus, were removed. The fish were weighed, cut into small pieces, and vacuum freeze-dried for 48 h. Subsequently, a freeze grinder was used to grind the dried fish into a fine powder. Exactly 2 g of the fish powder was weighed, fortified with isotope-labeled internal standards of the HOPs, and allowed to stand for 5 min. Methanol-acetonitrile (1∶1, v/v) was then added, followed by vortex mixing and ultrasonication. After centrifugation, the supernatant was transferred to a fresh tube. The extraction process was repeated twice and all extracts were combined. The extract was evaporated under a gentle nitrogen flow and redissolved in a mixture of ethyl acetate-cyclohexane (1∶1, v/v). The sample mixture was cleaned using gel permeation chromatography, and the eluate was collected and concentrated under a nitrogen flow. Sample residuals were reconstituted with water-methanol (1∶1, v/v) prior to instrumental analysis. Chromatographic separation was performed using an ACQUITY UPLC BEH C18 column (100 mm×2.1 mm, 1.7 μm). Water containing 2 mmol/L NH_4_Ac and acetonitrile were used as the mobile phases, and an optimized gradient elution program was applied. Isotope dilution and an internal standard method were used to quantify the HOPs. An electrospray ionization source operated in negative mode was applied to ionize the HOPs, and a full scan together with data-dependent acquisition (DDA) was applied for HRMS. Excellent linearities (*R*^2^>0.99) were obtained for all HOPs in the quantification range of 1.0-1000.0 ng/mL. The limits of quantification were 0.5 ng/g. The analytical method was validated using pooled fish samples fortified with HOP standards (4, 40, and 400 ng/g). The recoveries of the HOPs were in the range of 67.6%-133.8%, and the corresponding RSDs were 0.5%-15.6%. A total of 27 commercially available fish samples were analyzed using the developed method, and the results revealed the presence of HOPs in the fish, indicating the practicability of the method for real-world samples. The developed method is rapid, accurate, precise, and suitable for detecting HOPs in fish. This study provides a useful approach for environmental monitoring and food safety assurance by enabling the accurate and efficient analysis of HOPs in commonly consumed fish. Given increasing global concerns over HOPs, the method developed in this study will provide practical technical support for consumers aiming to reduce their exposure to and the adverse impacts of HOPs via fish.

卤代有机污染物(HOPs)包含有机氯农药(OCPs)、多氯联苯(PCBs)、多溴联苯醚(PBDEs)及全氟/多氟烷基物质(PFASs)等,多数具有持久性、生物积累性和潜在毒性,威胁人类健康和生态环境^[1,2]^。因此,HOPs的环境行为和生态毒理效应已成为全球关注和研究的重点。HOPs对环境健康的影响主要包括内分泌干扰、野生动物致癌、生育系统受损和其他健康危害^[3]^。近年来,四溴双酚A (TBBPA)和卤代咔唑(PHCZs)已被确定为海洋生态系统的常见污染物,在沉积物、海水和海产品中均被检出^[3,4]^。例如,生活在波罗的海区域的鱼类体内发现了含氯和含溴有机物^[5]^,在中国边缘海多种海产品样本(包括软体动物、鱼类和螃蟹)中已检测到TBBPA及其衍生物^[6]^。此外,研究发现,双酚A(BPA)在鱼类中含量约7.1~102.7 μg/kg^[7]^; Ashizuka等^[8]^研究了日本海产品中TBBPA的含量,实验一共研究了45份鱼类样品,这些样品分别来自不同的品种,其中29个鱼类样品中含有TBBPA,含量范围是0.01~0.1 ng/g ww(湿重);此外,来自各地的鱼罐头中BPA含量为2~59 ng/g ww^[9]^。由于HOPs具有较高的 ${K_{ow}}^{\left[10\right]}$ ,且不易溶于水,故这些化学物质(包括卤代咔唑、四溴双酚A及其类似物)可能会沿食物链转移累积^[11]^。例如,在美国旧金山湾的“双壳贝类-鱼(银汉鱼、条纹鲈鱼和白姑鱼) -斑海豹”的食物链中,已经发现了卤代咔唑的生物放大效应^[10]^。这些污染物会积累在水生生物体内,特别是长寿和高营养级生物,所以水产品中可能含有大量HOPs。作为蛋白质丰富的食物,鱼肉的食用是人体摄入HOPs的一个重要途径^[12]^。2018年全球鱼肉总产量已达到1.79×10^8^ t^[13]^,随着鱼肉产品食用量的增加,受污染鱼类产品亦会加剧影响人体健康。因此,食用鱼肉中HOPs的筛查分析和健康风险亟待研究。

目前文献报道的HOPs检测方法主要有气相色谱-质谱法^[14,15]^、液相色谱-质谱法^[16,17]^、傅里叶变换离子回旋共振质谱法(FT-ICR-MS)^[2]^和液相色谱-高分辨质谱法(LC-HRMS)^[18]^,其中液相色谱-质谱法简化了前处理步骤,能够提高准确性和灵敏度,目前广泛用于HOPs的检测。三重四极杆质谱定量准确,但是分辨率不足,而高分辨质谱则具有分辨率高和相对分子质量测定准确的优势,无需逐个优化目标化合物的检测参数,不受传统三重四极杆质谱检测化合物数量的限制,可通过提供离子的精确质荷比,实现复杂基质中目标化合物的准确定性^[19,20]^。鱼肉中脂肪含量高,基质复杂,而HOPs含量相对较低,会同时受到大分子蛋白质、脂肪和小分子氨基酸、有机酸、磷脂等的共同干扰^[21]^,实现多种HOPs的高通量筛查分析和同时检测存在挑战。

样品前处理的主要目的是将HOPs从复杂的基质中分离出来,并将目标物浓缩到足够的测量水平^[22]^。大部分HOPs具有不同程度的亲脂性,不容易直接通过溶剂进行萃取和净化,故选择采用凝胶渗透色谱法(GPC)净化处理。GPC是一种从色素、脂肪和蛋白质等大分子物质中分离化合物的方法,已被普遍用于环境有机污染物的净化^[23]^。GPC基于体积排阻原理将不同相对分子质量的物质进行分离,基质中的油脂、色素、生物碱、聚合物等高分子化合物会快速通过GPC柱,洗脱时间较短,相对分子质量较小的目标化合物则会缓慢通过GPC柱,通过控制填料凝胶孔径大小,调整干扰基质和目标化合物的洗脱时间,实现二者的有效分离^[24]^。

本研究基于HOPs的特性和鱼肉基质的特点,采用超声辅助萃取-凝胶渗透色谱净化联合超高效液相色谱-高分辨质谱(UPLC-HRMS)分析系统,建立了鱼肉中12种HOPs的同时检测方法。通过一级全扫描(Full scan)获得目标化合物的精确质量,同时结合数据依赖采集(DDA)二级扫描模式(dd-MS^2^)获得目标化合物的二级质谱数据。本研究实现了鱼肉中12种HOPs的准确、高效筛查分析,可为保障水产品安全和评估健康风险提供技术支持。相较于其他已报道的方法大多是专注于同一类结构相近物质的定量,本研究方法适用于定量多种结构差异大的HOPs。此外,GPC柱具备可重复使用的特性,有助于减少实验成本,安全环保,更加适用于批量样品的检测。

## 1 实验部分

### 1.1 仪器与试剂

超高效液相色谱仪(U300RSLC)、四极杆静电场轨道阱高分辨质谱仪(Q Exactive Focus)(美国Thermo-Fisher公司);涡旋振荡仪(VM-300,群安仪器实验有限公司);电子分析天平(ME2002E,上海梅特勒-托利多有限公司);移液器(德国Eppendorf公司);超声机(DTC-15J,鼎泰生化科技设备制造有限公司);离心机(H1650R,湖南湘仪实验室仪器开发有限公司);智能氮吹仪(TurboVap,瑞典Biotage公司);冷冻切割式研磨仪(NB-Wonbio-C,南北仪器有限公司);超纯水系统(Milli-Q IQ7000,美国Millipore公司)。

12种HOPs有机物标准品:氯霉素(CPL)、吡效隆(CPPU)、硫氯酚(BHL)、苄氯酚(CLF)购自德国 Dr. Ehrenstorfer公司;溴氯芬(BCP)购自美国PANPHY Chemicals Corporation公司;三氯生(TCS)、五氯酚(PCP)、对氯间甲酚(PCMC)、对氯间二甲苯酚(PCMX)购自美国Stanford Chemicals公司;2-碘苯酚(IPL)、四氯双酚A (TCBPA)购自上海麦克林生化科技股份有限公司;TBBPA购自上海阿拉丁生化科技股份有限公司。5种同位素内标:四溴双酚A-D_10_ (TBBPA-D_10_)、双酚A-D_16_ (BPA-D_16_)购自德国Dr. Ehrenstorfer公司;^13^C_6_-五氯酚(^13^C_6_-PCP)购自上海麦克林生化科技公司;三氯生-D_3_ (TCS-D_3_)、三氯卡班-D_4_ (TCC-D_4_)购自加拿大CDN Isotopes公司。

甲醇、乙腈、乙酸乙酯、环己烷(色谱纯,上海安谱实验科技股份有限公司),乙酸铵(纯度≥99%,上海麦克林生化科技股份有限公司),凝胶渗透玻璃色谱柱(400 mm×10 mm,广州芊荟化玻仪器有限公司),凝胶渗透色谱柱填料BiobeadsS-X3 (美国Bio-Rad Laboratories公司),聚四氟乙烯滤头(0.22 μm,天津津腾公司)。

### 1.2 标准溶液的配制

准确称量标准品,用甲醇充分溶解得到HOPs标准储备液,于-20 ℃冰箱保存。分别准确移取12种HOPs标准储备液于进样瓶中,用甲醇稀释,配制成质量浓度为10 μg/mL的混合标准工作液,涡旋混匀后于-20 ℃冰箱保存。根据实验需要,用甲醇配制成不同浓度的系列混合标准工作液,现用现配。

准确称量同位素内标标准品,加入甲醇溶解,得到内标储备液,于-20 ℃冰箱保存。分别精密移取5种同位素内标储备液于容量瓶中,用甲醇稀释,配制成质量浓度为5 μg/mL的同位素萃取内标工作溶液和同位素进样内标工作液,其中同位素萃取内标为三氯生-D_3_、三氯卡班-D_4_、四溴双酚A-D_10_。同位素进样内标为双酚A-D_16_和^13^C_6_-五氯酚。工作液于-20 ℃冰箱避光封口保存,现用现配。

### 1.3 样品前处理

将鲜活鱼表面用纯水冲洗,脱皮去骨除磷,取鱼肉部分称重、切成细块后真空冷冻干燥48 h,使用低温研磨机将其磨成粉末。准确称取2 g鱼肉粉末,加入10 μL 5 μg/mL萃取内标混合标准工作液,再加入15 mL甲醇-乙腈(1∶1, v/v),涡旋混匀;在20 ℃下超声提取20 min,以8000 r/min离心5 min,收集上清液,之后重复以上萃取步骤2次,合并3次萃取收集的上清液。经氮吹浓缩后,溶于1 mL乙酸乙酯-环己烷(1∶1, v/v)混合液,用注射器吸取混合液后过0.22 μm聚四氟乙烯滤膜,滤液过GPC柱净化,净化过程选用乙酸乙酯-环己烷(1∶1, v/v)作为洗脱液,取35 mL混合液淋洗,弃去前10 mL淋洗液,最终收集25 mL样品洗脱液,经氮吹浓缩至近干,加入甲醇定容至1 mL,准确加入10 μL 5 μg/mL进样内标混合标准工作液,混匀后过滤,待仪器分析。

### 1.4 分析条件

#### 1.4.1 色谱条件

色谱柱:ACQUITY UPLC BEH C18色谱柱(100 mm×2.1 mm, 1.7 μm);柱温:35 ℃;流动相:A为含2 mmol/L乙酸铵的水溶液,B为乙腈。梯度洗脱程序设置如下:0~0.2 min, 20%B; 0.2~8 min, 20%B~80%B; 8~10 min, 80%B~95%B; 10~12 min, 95%B; 12~12.1 min, 95%B~20%B; 12.1~15 min, 20%B。流速为0.25 mL/min,进样量为5.0 μL。

#### 1.4.2 质谱条件

采用电喷雾离子源(ESI),负离子模式;喷雾电压3.5 kV;毛细管温度320 ℃;加热器温度350 ℃;射频水平55%;鞘气流速45 arb;辅助气流速10 arb;扫描方式包括1次一级全扫描(分辨率为70000 FWHM), 1次数据依赖二级扫描(分辨率为35000 FWHM),全扫描范围均为*m/z* 70~1050,最大注入时间(IT)为100 ms,碰撞能量为10、30和50 eV。12种HOPs和5种内标物的保留时间和质谱参数见表1。

**表1:** 12种卤代有机污染物及5种同位素内标的化合物信息、保留时间及质谱参数

Compound	Formula	Retention time/min	Ion type	Measured accurate mass (*m/z*)	Theoretical exact mass (*m/z*)	Mass error/ 10^-6^ (ppm)	IS
Chloramphenicol (CPL)	C_11_H_12_Cl_2_N_2_O_5_	4.53	[M-H]^-^	321.00537	321.00505	0.996	TBBPA-D_10_
Pentachlorophenol (PCP)	C_6_HCl_5_O	5.78	[M-H]^-^	264.83704	264.83678	0.995	TBBPA-D_10_
Forchlorfenuron (CPPU)	C_12_H_10_ClN_3_O	6.34	[M-H]^-^	246.04419	246.04396	0.923	TCS-D_3_
*p*-Choro-*m*-cresol (PCMC)	C_7_H_7_ClO	6.65	[M-H]^-^	141.01137	141.01126	0.738	TCS-D_3_
2-Iodophenol (IPL)	C_6_H_4_IOH	6.63	[M-H]^-^	218.93132	218.93123	0.406	TBBPA-D_10_
4-Chloro-3,5-dimethylphenol (PCMX)	C_8_H_9_OCl	7.35	[M-H]^-^	155.02702	155.02691	0.671	TBBPA-D_10_
Bromchlorophen (BCP)	C_13_H_8_Br_2_Cl_2_O_2_	7.77	[M-H]^-^	424.81787	424.81749	0.902	TBBPA-D_10_
Clorofene (CLF)	C_13_H_11_ClO	8.79	[M-H]^-^	217.04269	217.04256	0.571	TCC-D_4_
Triclosan (TCS)	C_12_H_7_Cl_3_O_2_	9.72	[M-H]^-^	286.94427	286.94388	1.339	TCS-D_3_
Bithionol (BHL)	C_12_H_6_Cl_4_O_2_S	8.09	[M-H]^-^	354.87442	354.87403	1.087	TCS-D_3_
Tetrachlorobisphenol A (TCBPA)	C_15_H_12_Cl_4_O_2_	8.98	[M-H]^-^	364.94934	364.94891	1.168	TBBPA-D_10_
Tetrabromobisphenol A (TBBPA)	C_15_H_12_Br_4_O_2_	9.57	[M-H]^-^	542.74640	542.74571	1.271	TBBPA-D_10_
TBBPA-D_10_	C_15_H_2_D_10_Br_4_O_2_	9.53	[M-H]^-^	552.80939	552.80848	1.650	
TCS-D_3_	C_12_H_4_D_3_Cl_3_O_2_	9.69	[M-H]^-^	289.96280	289.96271	0.289	
TCC-D_4_	C_13_H_5_D_4_Cl_3_N_2_O	9.64	[M-H]^-^	316.99615	316.99587	0.863	
^13^C_6_-PCP	^13^C_6_HCl_5_O	5.79	[M-H]^-^	270.85703	270.85691	0.461	
BPA-D_16_	C_15_D_16_O_2_	6.47	[M-H]^-^	241.19600	241.19562	1.544	

### 1.5 数据处理

用Xcalibur软件采集、分析和处理检测结果和数据,用Origin 2021软件进行图表的绘制,用Excel进行数据统计和计算。

## 2 结果与讨论

### 2.1 质谱条件优化

质谱条件是影响HOPs前体离子和碎片离子响应强度的关键因素。本实验优化了电离模式和归一化碰撞能量。利用液相色谱-四极杆静电场轨道阱高分辨质谱对12种HOPs混合标准溶液进行正负离子扫描,得到一级全扫描质谱图,发现部分HOPs在正离子模式下无响应,且12种HOPs在负离子模式下响应值均较高,这是由于卤素是电负性较大的元素,在负离子模式下,分子中的卤素原子吸引周围的电子,容易形成负离子,而卤素原子连接的碳原子上和极性官能团上的氢原子会相对容易失去,从而形成[M-H]^-^。最终确定扫描模式为负离子模式,根据卤族元素具有不同丰度的同位素规律,选用天然丰度最高的离子作为定量和定性离子。

### 2.2 色谱条件优化

#### 2.2.1 色谱柱的选择

色谱柱的类型、填料和长度是影响目标化合物分离的重要因素。选择合适的色谱柱,目标物可以得到更好的分离,也可以提高方法的灵敏度和稳定性。本文比较了ACQUITY UPLC CSH (50 mm×2.1 mm, 1.7 μm)、ACQUITY UPLC BEH C18 (100 mm×2.1 mm, 1.7 μm)和ACQUITY UPLC HSS T3 (100 mm×2.1 mm, 1.8 μm) 3种色谱柱的分离效果。结果显示,ACQUITY UPLC BEH C18色谱柱对各物质的分离度最好,色谱信号响应最佳,因此采用该色谱柱分析鱼肉中的12种HOPs。

#### 2.2.2 流动相的选择

甲醇-水、乙腈-水体系是反相液相色谱的首选流动相体系,其中乙腈-水具有较强的洗脱能力,特别是有机相比例较低时,此优势更为明显。由于甲醇和乙腈的化学性质具有较大差异,甲醇属于质子性溶剂,而乙腈属于非质子性溶剂。而乙酸铵能够有效改善色谱峰形^[25]^,并且提高负离子模式下的离子化效率。实验比较了流动相分别为乙腈-水、甲醇-水(含2 mmol/L乙酸铵)、乙腈-水(含5 mmol/L乙酸铵)、乙腈-水(含2 mmol/L乙酸铵)的分离效果。结果表明,2 mmol/L的乙酸铵能够最大限度地增加目标化合物的响应值并改善峰形,而过高浓度的乙酸铵反而会降低目标化合物的离子化效率。而乙腈作为有机相时目标化合物的信号响应普遍比甲醇好,所以实验最终选择含2 mmol/L乙酸铵的水溶液和乙腈作为流动相。12种HOPs (800 ng/mL)和5种同位素内标(50 ng/mL)在最优实验条件下的提取离子流色谱图如图1所示。

**图1:**
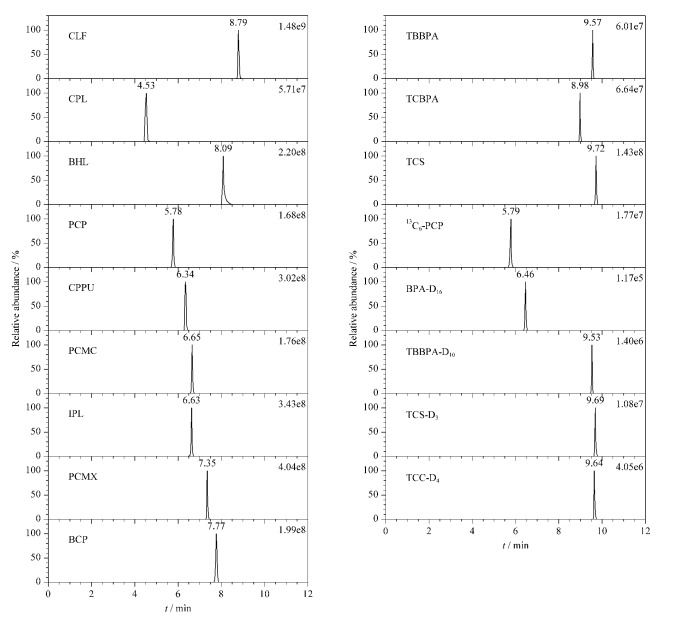
12种卤代有机污染物和5种同位素内标的提取离子流色谱图

### 2.3 前处理方法的优化

#### 2.3.1 提取溶剂的选择

有机污染物常用的提取溶剂有甲醇和乙腈。由于鱼肉中有较高含量的蛋白质和脂肪,在萃取过程中容易乳化而影响萃取效果,因此需要对鱼肉进行去除蛋白质和脂肪的处理以提高萃取效率。本实验比较了甲醇、乙腈、甲醇-乙腈(1∶1, v/v)对HOPs提取效果的影响。结果显示,相较于乙腈,甲醇与甲醇-乙腈(1∶1, v/v)具有较好的提取效果(见图2),考虑到鱼肉中蛋白质含量较高,提取溶剂中加入乙腈可以使蛋白质沉淀,后续净化更加容易,且混合溶剂具有更宽的溶解度范围,有助于提取不同极性的目标物。故采用甲醇-乙腈(1∶1, v/v)作为提取溶剂。

**图2:**
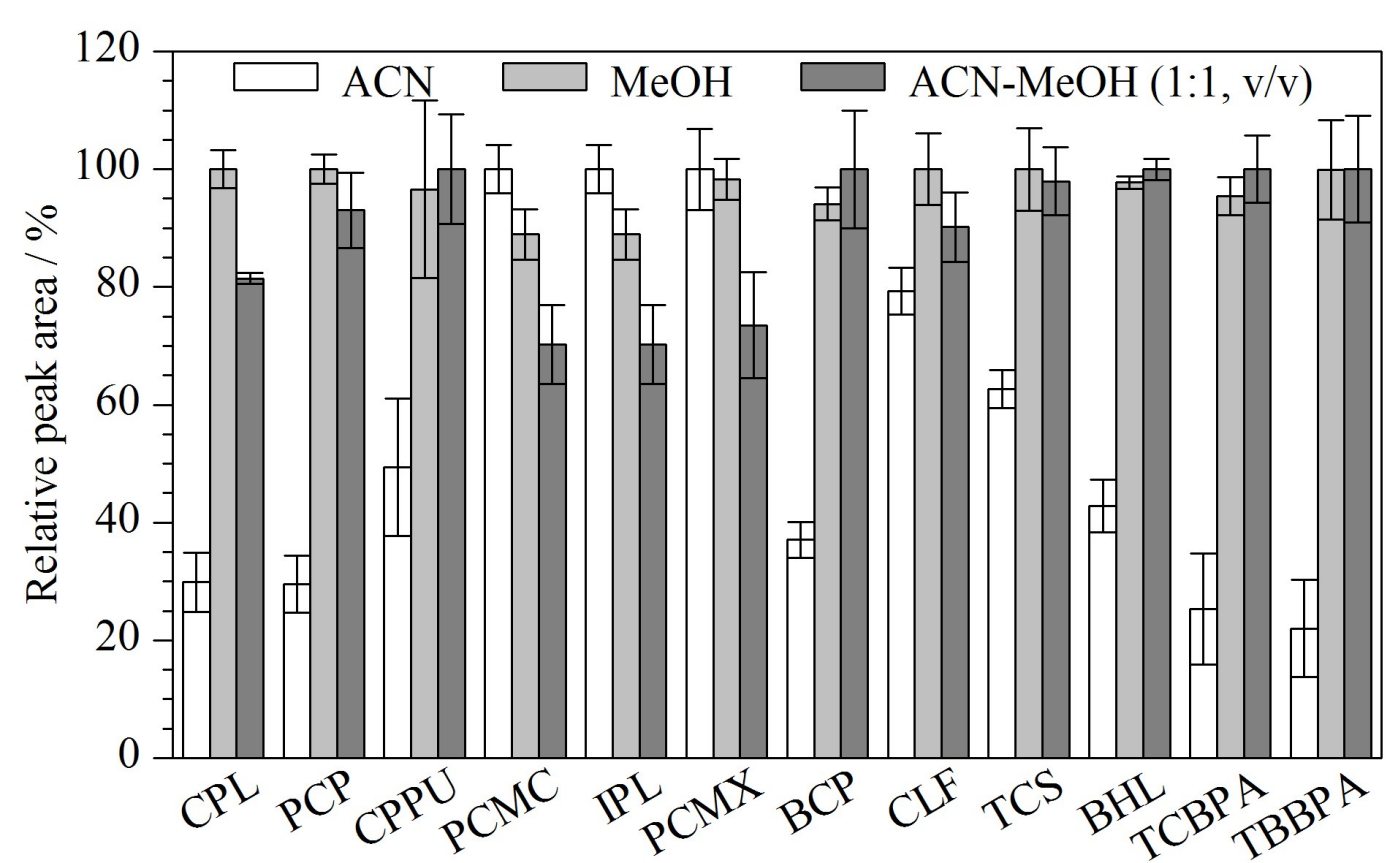
采用不同提取溶剂时12种HOPs的相对峰面积(*n*=3)

#### 2.3.2 提取溶剂体积的选择

本实验对比了单次提取溶剂体积为10、15、20 mL时12种HOPs的相对峰面积(见图3)。结果表明,目标物随着提取溶剂体积的增加,相对峰面积呈先增加后减小的趋势,这可能是因为随着提取剂体积增加,提取到的杂质也随之增加,基质效应加剧,从而影响目标物检测。因此,最终单次提取溶剂体积确定为15 mL。

**图3:**
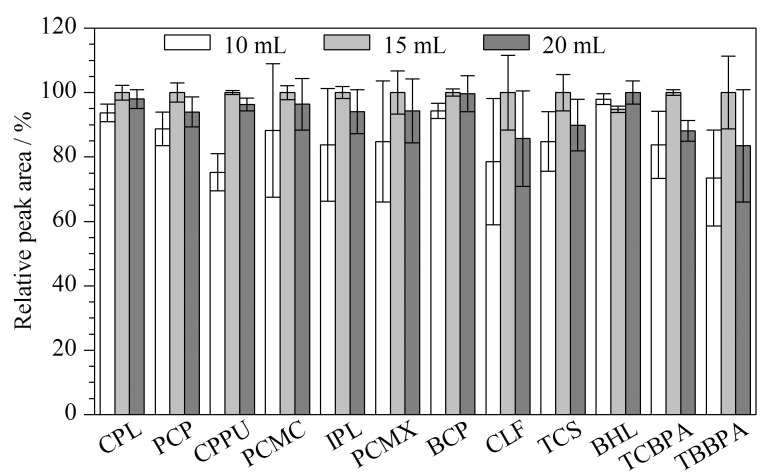
采用不同提取体积时12种HOPs的相对峰面积(*n*=3)

#### 2.3.3 洗脱液体积的选择

GPC是一种温和的样品净化方法,无强酸强碱刺激,主要依据物理分离原理除去大分子物质,具体过程为油脂等大分子物质会快速通过色谱柱,洗脱时间短,分子质量较小的物质,洗脱时间长,从而使油脂等干扰基质与目标物质分离^[26]^。由于不同分子质量的物质流出时间不固定,洗脱体积亦有区别,故以1 mL为单位单独收集每份洗脱液,氮气吹扫近干后用甲醇定容测试,根据每毫升各物质的峰面积来推算合适的洗脱体积,并拟合成流出曲线(见图4)。结果显示,12种HOPs都在10~20 mL完全洗脱,为了减少交叉污染的风险和可能残留在柱子上的前一次分析的残留物,故选择25 mL的洗脱体积。

**图4:**
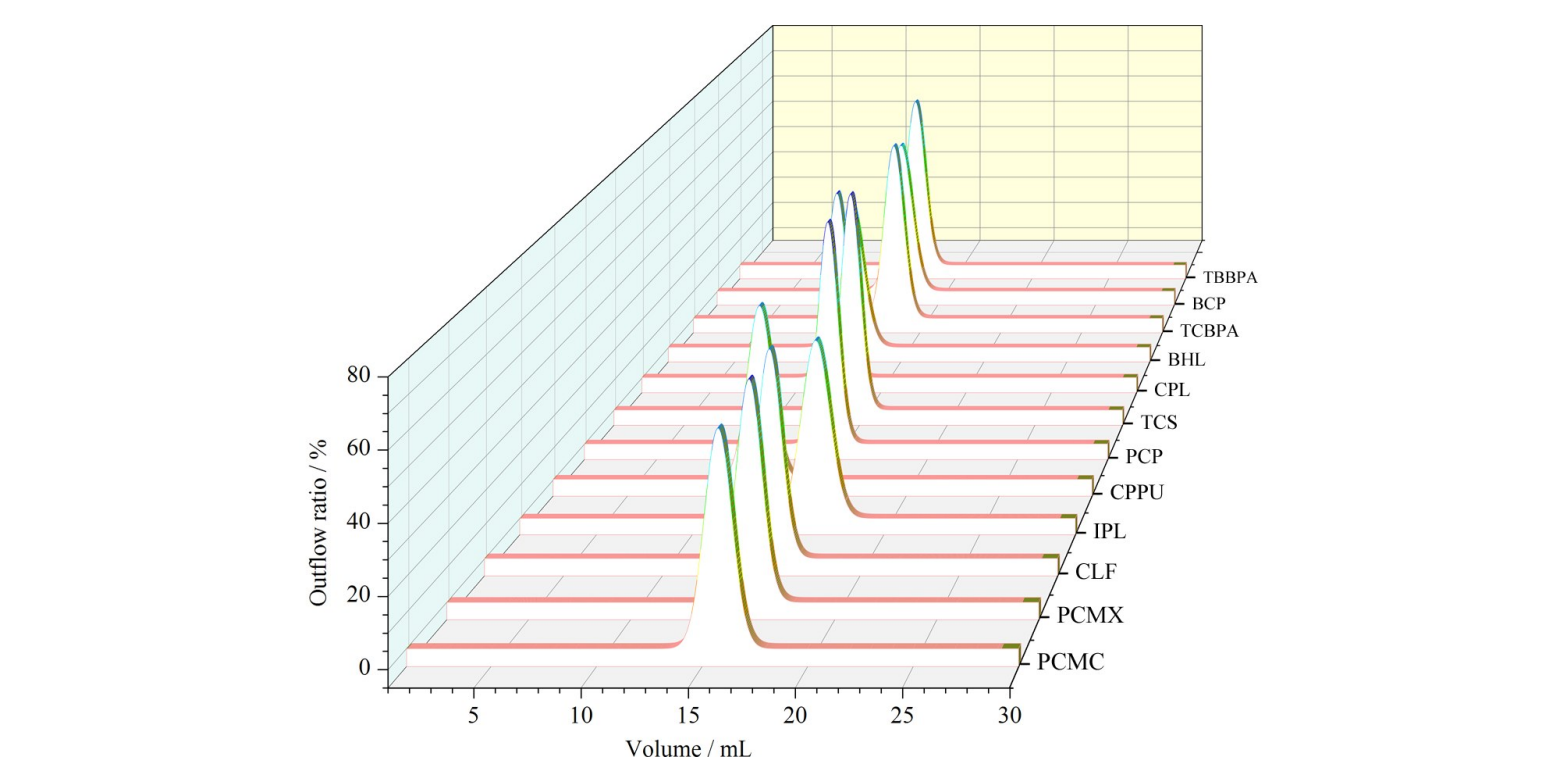
12种卤代有机污染物过凝胶渗透色谱柱的流出曲线

### 2.4 方法学验证

#### 2.4.1 线性范围与定量限

对不同浓度的标准溶液进行分析,以化合物的峰面积与萃取内标物峰面积之比为纵坐标,质量浓度之比为横坐标,进行线性回归拟合,得到线性方程和相关系数(*R*^2^)。结果表明,12种HOPs在1.0~1000.0 ng/mL范围内线性良好,相关系数均大于0.99。由于Q Exactive Focus静电场轨道肼高分辨质谱仪具有出色的分辨率,大部分目标物即使在较低浓度下信噪比也接近于无限大,使得传统的基于3倍信噪比确定检出限的方法不再适用。在这种情况下,为了更准确地界定分析的灵敏度,采用定量限作为评估指标,将标准曲线下限作为定量限^[27]^。由于实验中单个样品量为2 g,计算得到方法的LOQ为0.5 ng/g。

#### 2.4.2 回收率和精密度

在鱼类样品中分别添加3个水平(400、40、4 ng/g)的12种HOPs混合标准溶液进行加标回收试验,按照优化的前处理条件进行提取和净化,每个水平制备3个平行样,计算平均加标回收率和相对标准偏差,并通过加标样品与质控样品中进样内标峰面积之比再次校正目标物的峰面积以减少在定容和进样时产生的微小误差。结果显示,12种HOPs的平均加标回收率为67.6%~133.8%,精密度为0.5%~15.6%(见表2),基本满足定量分析要求。

**表2:** 12种卤代有机污染物的线性范围、回归方程、加标回收率和精密度(*n*=3)

Compound	Linear range/(ng/mL)	Regression equation	Spiked level/(ng/g)	Recovery/%	RSD/%
CPL	1.0-1000.0	*Y*=0.457+0.079*X*	400	91.8	0.6
			40	128.3	2.1
			4	82.0	3.7
PCP	1.0-1000.0	*Y*=0.038+0.051*X*	400	80.5	0.9
			40	116.2	1.9
			4	96.1	2.8
CPPU	1.0-1000.0	*Y*=5.356+0.218*X*	400	84.9	0.9
			40	102.0	1.2
			4	68.9	4.5
PCMC	1.0-1000.0	*Y*=-4.165+1.251*X*	400	107.1	7.8
			40	102.5	0.5
			4	133.8	0.9
IPL	1.0-1000.0	*Y*=8.471+0.914*X*	400	107.3	4.3
			40	118.4	3.2
			4	114.6	5.1
PCMX	1.0-1000.0	*Y*=-4.810+0.374*X*	400	106.1	8.1
			40	108.0	7.5
			4	101.6	5.7
BCP	1.0-1000.0	*Y*=0.017+0.065*X*	400	102.0	5.6
			40	105.4	1.9
			4	112.0	8.1
CLF	1.0-1000.0	*Y*=1.484+0.366*X*	400	105.6	6.3
			40	102.3	12.9
			4	71.1	8.1
TCS	1.0-1000.0	*Y*=0.620+0.079*X*	400	99.8	1.5
			40	106.1	12.5
			4	108.2	0.6
BHL	1.0-1000.0	*Y*=3.873+0.414*X*	400	101.8	5.0
			40	98.2	3.3
			4	96.5	9.2
TCBPA	1.0-1000.0	*Y*=-2.501+0.209*X*	400	99.8	5.6
			40	105.5	7.2
			4	67.6	3.7
TBBPA	1.0-1000.0	*Y*=-0.021+0.002*X*	400	104.3	9.9
			40	87.3	15.6
			4	107.1	2.4

*Y*: peak area ratio of the analyte to extract interal standard; *X*: mass concentration ratio of the analyte to extract internal standard.

### 2.5 实际样品的检测

购买来自广州、东莞、佛山、中山、惠州、肇庆、清远各城市地区常见市售各类鱼肉样品共27份,用本实验建立的检测方法进行分析检测。检测结果表明,实际样品中检出多种HOPs,详细数据如表3所示。除PCMC、BCP、BHL和TCBPA外,其余HOPs均有检出,其中IPL检出率最高,达51.9%,检测含量最高的为PCP,含量为4.52 ng/g。PCP是一种广泛用于防腐剂或者木材保护的化学品,而IPL则是抗菌肥皂、消毒液的主要成分,这可能导致其在工业区域或生活区域水体中的含量较多。鱼类通过呼吸和食物摄取水中的物质,导致其在体内积累。

**表3:** 实际样品中12种卤代有机污染物的含量

Sample No.	CPL	PCP	CPPU	PCMC	IPL	PCMX	BCP	CLF	TCS	BHL	TCBPA	TBBPA
1	<LOQ	ND	ND	ND	0.50	ND	ND	ND	ND	ND	ND	ND
2	ND	ND	ND	ND	ND	ND	ND	ND	ND	ND	ND	ND
3	ND	ND	ND	ND	0.51	ND	ND	ND	ND	ND	ND	ND
4	<LOQ	3.58	<LOQ	ND	1.10	<LOQ	ND	<LOQ	ND	ND	ND	ND
5	1.22	4.52	<LOQ	ND	0.65	<LOQ	ND	<LOQ	ND	ND	ND	ND
6	<LOQ	3.15	<LOQ	ND	1.05	<LOQ	ND	<LOQ	<LOQ	ND	ND	1.54
7	ND	ND	ND	ND	ND	ND	ND	ND	ND	ND	ND	ND
8	ND	ND	ND	ND	0.51	0.87	ND	ND	ND	ND	ND	3.67
9	ND	ND	ND	ND	<LOQ	<LOQ	ND	ND	ND	ND	ND	ND
10	ND	ND	<LOQ	ND	<LOQ	ND	ND	ND	<LOQ	ND	ND	ND
11	<LOQ	ND	<LOQ	ND	ND	ND	ND	<LOQ	<LOQ	ND	ND	0.84
12	ND	1.76	ND	ND	ND	ND	ND	ND	ND	ND	ND	ND
13	ND	ND	ND	ND	<LOQ	<LOQ	ND	ND	ND	ND	ND	ND
14	0.59	ND	ND	ND	ND	ND	ND	ND	ND	ND	ND	ND
15	ND	1.59	ND	ND	ND	ND	ND	ND	ND	ND	ND	ND
16	<LOQ	1.67	ND	ND	ND	ND	ND	ND	ND	ND	ND	ND
17	<LOQ	ND	ND	ND	ND	ND	ND	ND	ND	ND	ND	ND
18	ND	1.49	ND	ND	<LOQ	ND	ND	ND	ND	ND	ND	<LOQ
19	<LOQ	1.52	<LOQ	ND	0.51	<LOQ	ND	<LOQ	<LOQ	ND	ND	ND
20	ND	ND	<LOQ	ND	<LOQ	<LOQ	ND	ND	ND	ND	ND	ND
21	1.16	1.73	ND	ND	ND	<LOQ	ND	ND	ND	ND	ND	ND
22	ND	ND	ND	ND	ND	<LOQ	ND	ND	ND	ND	ND	<LOQ
23	ND	1.77	ND	ND	ND	ND	ND	ND	ND	ND	ND	0.74
24	ND	ND	<LOQ	ND	<LOQ	ND	ND	ND	ND	ND	ND	ND
25	ND	ND	ND	ND	ND	ND	ND	ND	ND	ND	ND	ND
26	ND	ND	ND	ND	<LOQ	<LOQ	ND	ND	ND	ND	ND	ND
27	ND	ND	<LOQ	ND	ND	ND	ND	ND	ND	ND	ND	ND

ND: not detected.

## 3 结论

本研究成功建立了通过超声萃取-GPC净化联合UPLC-HRMS同时测定食用鱼肉中12种HOPs的快速筛查及精准定量的检测方法。通过甲醇-乙腈混合溶剂体系和超声辅助萃取,结合低温高速离心及凝胶渗透色谱净化,实现了目标分析物的高效萃取和净化。采用全扫描加数据依赖采集模式,对目标分析物进行准确定量分析。方法验证结果表明所建立分析方法具有良好的准确度、重复性和灵敏度。本研究为肉类食品中HOPs有机污染物的防控提供了可靠分析方法,为市场监管部门和相关食品经营企业提供了有效的技术支撑,有助于保障食品安全和消费者健康。

## References

[1] Tang C M, Zhu Y Z, Liang Y Y, et al. Environ Sci Technol, 2023, 57(3): 1378

[2] Tang C M, Chen G S, Jiang B, et al. Anal Chim Acta, 2022, 1204: 339618 35397908 10.1016/j.aca.2022.339618

[3] Zhu H H, Zheng M G, Zheng L, et al. Mar Pollut Bull, 2019, 146: 393 31426173 10.1016/j.marpolbul.2019.06.078

[4] Liu K, Li J, Yan S J, et al. Chemosphere, 2016, 148: 8 26800486 10.1016/j.chemosphere.2016.01.023

[5] Wu Y, Tan H L, Sutton R, et al. Environ Sci Technol, 2017, 51(4): 2038 28112952 10.1021/acs.est.6b05733

[6] Liu A F, Qu G B, Yu M, et al. Environ Sci Technol, 2016, 50(8): 4203 10.1021/acs.est.5b06378 27008063

[7] Munguia-Lopez E M, Gerardo-Lugo S, Peralta E, et al. Food Addit Contam, 2005, 22(9): 892 10.1080/02652030500163674 16192075

[8] Ashizuka Y, Nakagawa R, Hori T, et al. Mol Nutr Food Res, 2008, 52(2): 273 10.1002/mnfr.200700110 18246587

[9] Podlipna D, Cichna-Markl M. Eur Food Res Technol, 2006, 224(5): 629

[10] Karl H, Ruoff U. Chemosphere, 2007, 67(9): S90 10.1016/j.chemosphere.2006.05.121 17223169

[11] Silva A B, Bastos A S, Justino C I L, et al. Anal Chim Acta, 2018, 1017: 1 29534790 10.1016/j.aca.2018.02.043

[12] Chessa G, Cossu M, Fiori G, et al. Chemosphere, 2019, 228: 249 31035162 10.1016/j.chemosphere.2019.04.046

[13] Wang Q. Fishery Information & Strategy, 2022, 37(3): 237

[14] Gao Z Y, Jiang L B, Deng S G, et al. Journal of Food Safety & Quality, 2023, 14(4): 112

[15] Tang C M, Chen G S, Liang Y T, et al. Anal Chim Acta, 2022, 1222: 340171 35934429 10.1016/j.aca.2022.340171

[16] Xu H Y. [MS Dissertation]. Qingdao: Qingdao University, 2022

[17] Wu J. [MS Dissertation]. Wuhan: Changjiang University, 2023

[18] Tang C M, Zheng R F, Zhu Y Z, et al. Environ Sci Technol, 2023, 57(44): 17099 10.1021/acs.est.3c04239 37878998

[19] Ma J M, Sun L, Cao M R, et al. Food Science, 2020, 41(4): 273

[20] Qi C Y, Xu X L, Guo W, et al. Food Science, 2022, 43(4): 315

[21] Li J, Ju X, Wang Y L, et al. Chinese Journal of Chromatography, 2023, 41(7): 610 10.3724/SP.J.1123.2022.10010 PMC10311622 37387282

[22] Wang Y, Li J, Ji L, et al. Molecules, 2022, 27(7): 2160 35408558 10.3390/molecules27072160 PMC9000397

[23] Wu Y, Moore J, Guo J H, et al. J Chromatogr A, 2016, 1434: 111 26818240 10.1016/j.chroma.2016.01.036

[24] Li S J, Dong M, Xu H, et al. Chinese Journal of Chromatography, 2014, 32(2): 157 10.3724/sp.j.1123.2013.10016 24822450

[25] Li R, He C M, Yang L Q, et al. Chinese Journal of Chromatography, 2017, 35(8): 808 10.3724/SP.J.1123.2017.03035 29048814

[26] Xiong S S. [MS Dissertation]. Guangzhou: University of Chinese Academy of Sciences (Guangzhou Institute of Geochemistry, Chinese Academy of Sciences), 2017

[27] U. S. Food and Drug Administration. Bioanalytical Method Validation, Guidance for Industry. (2018-05-24). http://www.fda.gov/AnimalVeterinary/GuidanceComplianceEnforcement/GuidanceforIndustry/default.htm http://www.fda.gov/AnimalVeterinary/GuidanceComplianceEnforcement/GuidanceforIndustry/default.htm

